# Adaptive Smart Technology Use: The Need for Meta-Self-Regulation

**DOI:** 10.3389/fpsyg.2017.00298

**Published:** 2017-03-02

**Authors:** Theresa Schilhab

**Affiliations:** Future Technologies, Culture and Learning, Danish School of Education, University of AarhusCopenhagen, Denmark

**Keywords:** smart technology, reading, novelty effects, self-regulation, automatization, attention regulation, smartphones, tablets

## Introduction

Today, smart technology in the form of tablets and smartphones is a cherished tool for most people. Instant online access that allows for extensive interacting on social media, texting, playing video games and music, checking for news and weather has turned smart technology into “an integral part of the lives of all ages worldwide” (Samaha and Hawi, 2016, p. 321). The multi-functional nature of smart technology makes it attractive as a tool for learning and education (Kucirkova, 2014; Schilhab, 2017a) leading, however, to noticeable changes in affordances and embodiment, and consequently learning (Mangen and Schilhab, 2012)^1^.

For instance, reading scholars increasingly find that changing the physical reading platform (from a printed book to a digital screen) leads to marked alterations in comprehension of the text read. They point to factors related to the affordance of the reading device such as haptics e.g., perception through touch (Mangen and Kuiken, 2014) and lighting conditions (e.g., Benedetto et al., 2013) as aspects undergoing a significant change which result in a reduced learning outcome. This observation is corroborated by studies probing for accompanying metacognitive processing that show less accurate prediction of performance and more erratic study-time regulation when reading on screen versus on paper (Ackerman and Goldsmith, 2011).

Such effects on literary reading are not agreed upon unanimously. Some researchers emphasize our “native biological plasticity” that among other things entails “bodily reconfiguration” Clark (2007 p. 263) and major “re-embodiment” (Ihde, 2010) when describing human cognition in relation to smart technology use. The argument asserts that the reduced comprehension when reading on screen is a novelty effect in the sense that subjects are proficient print-readers while still lacking in screen expertise (Hayler, 2015). Over time, people will adjust to the affordances of the new devices and the comprehension issues apparent today will evaporate as screen reading abilities are simultaneously refined and technology has co-evolved for this specific task.

In so far as screen use is tool-use, the proposed plasticity- and embodiment perspective prevalent in human-technology interaction studies seems pertinent. The question remains, however, if prolonged exposure and subsequent development of embodied skills is all it takes for humans to adapt to the affordances of smart technology. A relevant objection to the novelty claim could be that the comprehension issues associated with screen reading exemplifies a need to go beyond automatic embodiment processes and conceptualize the specifics of the mental processes that account for our adaptation to the environment.

In the following article, I unfold why automatic skill learning may not be an exhaustive answer to the affordances provided by smart technology. First, I discuss what characterizes smart technology tools from the perspective of attention to better determine significant features of the interaction and what adaptive processes would entail. I conclude that section by proposing that the interaction calls for self-regulation. Second, I discuss a socially mediated mechanism that seems especially supportive in the building of such capacities necessary for environmental coping in general and smart technology use in particular.

## Smart technology and attention

Part of the controversy over reading performance in literature studies stems from the inevitable complex relations we form with the environment (e.g., Jin et al., 2015). On the one hand, when musicians interact with their instruments like pianos for instance, or high jumpers with their bars, they adapt to factors of the interaction that all else being equal manifest little variation (e.g., Jabusch et al., 2009). Crudely put, to adapt to similarities of tasks consists among other things in strengthening the overall connection among neurons in the neural correlate to increase the signaling efficiency (Draganski et al., 2004; Jäncke, 2009). A skilled performance partly evolves because of strengthening of automatic bottom-up processes elicited by the repetition of particular elements in the task (Maguire et al., 2006).

This may be exemplified by an fMRI study on how processes underlying imagery differ among novices and experts in a complex motor skill (the high jump) showing considerable divergence with respect to the involvement of motor areas such as the supplementary motor area (SMA) and primary motor cortex (Olsson et al., 2008). Subjects were asked to imagine the performance of a full jump, with special emphasis on certain stages, such as take-off or clearing the bar. Novices who did not have previous experience of the high jump showed more activation in areas that suggested that they took an external view of the task (watching the jumps from without as if out of the body) possibly, because their previous experiences with high jumps were primarily as spectators to high jumps. Thus, the activation of SMA, which is suggested to be responsible for internally guided actions both while executed and imagined, was lower in novices than it was in expert high jumpers. Following the authors, the use of an internal perspective during motor imagery of a complex skill depends on well-established motor representations of the skill before these can translate into a motor/internal pattern of brain activity (Olsson et al., 2008, p. 5).

On the other hand, we have interactions that are characterized by intrinsic variability. In the course of evolution, our coping with such seemingly unstable factors has been optimized by development of fine-tuned attentional resources to help us focus on particular aspects of the environment (e.g., Kaplan and Berman, 2010). Why is this of importance when dealing with our possibilities for adapting to smart technology? The reason is that part of our interaction with smart phones and tablets when for instance reading is defined especially by the erratic factors that elicit our vigilance (e.g., Chun et al., 2011). In fact, the multi-functional affordance of the tool may itself drag attentional resources diminishing the attention we normally allocate to reading in order to comprehend the text (Wolf and Barzillai, 2009, for a recent discussion on stable and variable affordances of relevance to the present discussion, see Sakreida et al., 2016). The appeal for diverse activities such as checking for emails, surfing the internet, or tapping into social media while reading is in effect even if notifications and various alerts are deliberately turned off. The mere awareness of putative distraction may reallocate attention from comprehension processes (Przybyliski and Weinstein, 2013; Schilhab et al. submitted).

## The need for intentionality

Besides the smart-tool features, smart technology affords instant distraction and gratification, such as watching videos, gaming or establishing social contact online, which drags attention from other tasks. Thus, those smart technology interactions that drag attentional resources are not prone to implicate automatized processes in the procedural sense of the term. Hence, appropriate adaptation to smart technology as a tool needs to go beyond mere embodiment and to involve some kind of attention regulation as suggested by studies emphasizing risks of addiction in connection to increased smart technology (Wei et al., 2012; Tarafdar et al., 2013). When people check for messages and updates not because they need to, but out of habit (Lee et al., 2014) and are deeply attracted to the device even in the company of others (Radesky et al., 2014), social relations may become challenged (Turkle, 2015).

But how do we cultivate attention regulation?

Attention regulation is closely connected to executive functions (EFs), which refer to an assembly of functions in use when we concentrate and think. The core functions are inhibition, working memory, and cognitive flexibility and form the basis for “higher-order” EFs such as reasoning, problem solving, and planning (Diamond, 2013).

Working memory is the function that holds back information in mind to be manipulated. It is involved in making sense of linguistic information, to derive a general principle, and acknowledge novel relations among old ideas (Diamond and Lee, 2011).

Inhibition refers to control of behavior, such as when inhibiting habitual responses and resisting short-sighted temptations such as leaving a task unsolved or incomplete. Inhibition is exercised in attention regulation to corroborate focused and directed attention and in emotional self-control.

It is EFs that allow us to perform “offline” tasks (Wilson, 2002), i.e., tasks that do not depend on information from the environment but on sustained imagery (e.g., Schilhab, 2015a), while fencing off disturbing stimulations (Vanhaudenhuyse et al., 2010). During reading for instance, the active construction of meaning (Wolf and Barzillai, 2009) involves maintenance of competing interpretations until a final solution to the developing understanding is found (e.g., Smallwood et al., 2008).

On the other hand, cognitive flexibility manages perspective change, for instance switching between different aspects, thinking outside the box, and understanding the perspective of other people (Schilhab, 2015a).

Overall, EFs have been linked to better academic skills, better quality of life, and improved self-assessment and are to some extent trainable (Diamond, 2012).

## The social dimension of the “inner” system

An assertion of attention regulation implicitly assumes that there exist mental operations beyond those evolving as bottom-up embodiment bound adaptations to the environment (though kinds of attention regulation may be closely connected to the action-perception cycle, see for instance Jin and Lee, 2013 and Jin et al., 2015 for the discussion of how the training of *Kih* may lead to affordance-control).

Here, I suggest that, although attention regulation exists as a potential operation of the mind of the individual, its actualization depends on social interaction in a certain kind of conversational exchange (Schilhab, 2015b). Ordinary discourse, in which participants exchange information on the fly, may happen at a superficial level without substantial attentional investment on the part of the interlocutors. Everyday exchanges of words for instance need not recruit focal thought to satisfy the purpose of a dialogue. For conversation to result in acquisition of abstract knowledge, the more knowledgeable (the parent or care taker) must take responsibility to create mutual comprehensibility in the conversation by assessing the perspective of the learner and fill in the gaps to ensure coherence of the emerging conversation (a condition salient also in Vygotsky's zone of proximal development e.g., Hasse, 2014; see also Schilhab, 2015b, 2017b). Learning about abstract referents one has had no direct experience with places a different stress on the ability to sustain understanding. To convey abstract knowledge, the interlocutor will need to establish metaphors or phrases that immediately capture the concrete meaning of the abstract knowledge. Just as the adult in ostensive learning furnishes the immediate environment, for instance holding up a cup, pointing to the cup and exclaiming “cup” (e.g., Pulvermüller, 2012), the interlocutor furnishes the world that is off-line. He or she seeks mutual comprehensibility and makes mental tableaus that are thought to match the understanding of the child. In concrete language acquisition, interlocutors merely point to the referent of the conversation, whereas in abstract language acquisition, the interlocutor points by using words.

Thus, the mechanisms that lead to understanding fundamentally change. The cognitive efforts behind this process are comprehensive and advanced and include mastering an attentional switch from monitoring external stimulation to the internal “stream of consciousness” (e.g., Dennett, 1992).

Language elicited imagination depends on a certain degree of linguistic competence and is therefore likely to emerge relatively later in language acquisition. Moreover, for the ability to fully develop it is crucial to have emphatic interlocutors. My assertion is that a subject's abilities to acquire abstract knowledge and with that become trained in monitoring the internal stream of consciousness evolve most readily through careful guidance and therefore may in fact vary noticeably as an effect of a “master.”

## Final remarks

Some of the reported side effects of smart technology employment referred to by contemporary research, such as novelty effects, will definitely vanish when users become more proficient. Here, the embodiment processes work in response to the immediately present environment. However, combating distractors, which often operate as attention-grabbers, inherent to the affordances of smart technology calls for cognitive meta-processes elicited independently of the interaction with smart technology. As with any addictive “substance” that modern Western life has admitted almost unrestricted access to, such as calories, sessile life, alcohol, cholesterol, or on a larger scale fossil fuels, it is up to the individual to evolve a well-functioning, albeit cognitively exhausting, self-control. Though many avenues to achieve this ability are open, I suggest that the individual may quite effectively be gently nudged in the right direction by engaging in deep conversations with interlocutors. Mental mechanisms central to mediating understanding of what may not be concrete or present, simultaneously enhance the mechanisms we need in order to appropriately adapt to smart technology.

### Conflict of interest statement

The author declares that the research was conducted in the absence of any commercial or financial relationships that could be construed as a potential conflict of interest.

## Footnotes

^1^Kiverstein and Rietveld (2015, p. 709), affordances are ”the multiple possibilities for action that stand out as relevant for an individual in a particular situation because of their needs and concerns”. In this paper I compare those affordances that we address using'skilled intentionality' (ibid.) and affordances that attract our conscious attention. See however 1979/1986 for the original reading of the term.
